# Increased PPARD Expression May Play a Protective Role in Human Lung Adenocarcinoma and Squamous Cell Carcinoma

**DOI:** 10.1155/2022/9414524

**Published:** 2022-03-15

**Authors:** Yong Zhu, Yedong Mi, Zhonghua Qin, Xuewei Jiang, Yibo Shan, Kamil Kural, Guiping Yu

**Affiliations:** ^1^Department of Thoracic Surgery, The 9th People's Hospital of Suzhou, Suzhou, Jiangsu Province 215200, China; ^2^Department of Cardiothoracic Surgery, Jiangyin People's Hospital Affiliated to Nantong University, No. 163 Shoushan Rd, Jiangyin, Jiangsu Province 214400, China; ^3^School of Systems Biology, George Mason University, Manassas, VA 20110, USA

## Abstract

Peroxisome proliferator-activated receptor-*δ*, encoded by gene *PPARD*, is overexpressed in a majority of human lung cancer subtypes, but its role in the tumor progression remains poorly understood. We have analyzed the expression of *PPARD* in lung adenocarcinoma (LA) and squamous cell carcinoma (LSCC) datasets. The potential roles of *PPARD* in the pathological development of LA and LSCC were explored through literature-based pathway analysis and pathway enrichment analysis. In all LA datasets (*N* = 11) and in seven out of nine LSCC studies, the levels of *PPARD* were increased as compared to control tissues (log-fold changes were 0.37 ± 0.20 and 0.10 ± 0.37 for LA and LSCC, respectively). On average, the expression levels of *PPARD* in LA were higher than those in LSCC (*p* = 0.036). Pathway analysis showed that the overexpression of PPARD might play both positive and negative roles in the development of both LA and LSCC. Specifically, *PPARD* inhibits seven LSCC promoters and seven LA promoters and activates one LSCC inhibitor and another LA inhibitor. However, PPARD also activates six and one promoters of LA and LSCC, respectively, which would facilitate the development of LA/LSCC. Our results suggested a mixed role of PPARD in LA/LSCC, which may add new insights into the understanding of the PPARD-lung cancer relationship.

## 1. Introduction

Lung carcinoma (LC) is a leading cause of cancer death worldwide [1]. Annually, lung cancer kills nearly 1.8 million people, more than breast, prostate, pancreatic, and colorectal cancers combined [2]. Lung cancer has been divided into two main histological types, namely, small-cell lung carcinoma (SCLC) and non-small-cell lung carcinoma (NSCLC) [3]. The latter is further subdivided into lung adenocarcinoma (LA), which comprises around 40% of all LC [1], and lung squamous cell carcinoma (LSCC) that accounts for about 30% of all lung cancer [1, 4].

Several previous studies suggested a strong association between the expression levels of peroxisome proliferator-activated receptor ß/d- (PPARD-) encoding gene and human lung cancer [5–8]. A majority of the studies found that *PPARD* is overexpressed in lung cancer [5, 8, 9], with one recent study pointing at the association of high expression levels of this gene with a worse prognosis [10]. However, the role that PPARD plays in the pathophysiology of lung cancer is far from being clear. Even if some studies unequivocally point at PPARD as a lung cancer-promoting gene [8], others suggest that ligand-driven activation of PPARD may suppress the growth of lung cancer [6] by inhibiting inflammation [7].

To facilitate our understanding of the role of the PPARD-encoding gene in lung cancer, we explored its activity of PPARD in expression dataset profiling major subtypes of lung cancer, namely, LA and LSCC, and an influence of the increase in *PPARD* expression on the pathophysiology of LA and LSCC. We confirmed that in most cases of LA and LSCC, the expression of PPARD is increased, while its function may either enhance or suppress the proliferation of lung cancer depending on tissue context.

## 2. Method

The workflow of this study contains two significant sections. First, we collected publicly available gene expression datasets to explore and compare the expression changes of PPARD in the case of LA or LSCC. Then, we conducted large-scale literature data mining to build pathways connecting PPARD to LA or LSCC, revealing the potential role of PPARD in the pathological development of LA and LSCC.

### 2.1. Collection of Twenty Expression Datasets

To explore the expression activity of PPARD expression in LA and LSCC, we collected all available expression datasets within the Gene Expression Omnibus (https://www.ncbi.nlm.nih.gov/geo/). Keywords “lung adenocarcinoma” and “lung squamous cell carcinoma” were used for the dataset search. The data selection criteria were as follows: (1) the organism is Homo sapiens; (2) the data type is RNA expression by array; (3) the study has case vs. healthy control comparison; (4) the dataset and its format files are publically available; and (5) the datasets and its corresponding format files are publicly available. From each dataset, expression data for the healthy controls and LA/LSCC patients were extracted and reanalyzed.

### 2.2. PPARD Expression Analysis

To explore the PPARD expression within each independent study, instead of estimating an averaged expression value from all studies, we calculated and compared the PPARD expression log-fold change (LFC) for each dataset in the case of LA/LSCC compared to healthy controls. Multiple linear regression analysis has been conducted to explore the influence of multiple potentially influential factors on PPARD in the case of LA/LSCC, including sample population region (country), sample size, and sample profile collection date. Additionally, ANOVA has been used to compare the difference in expression patterns of PPARD between LA and LSCC cases.

### 2.3. PPARD-Driven Pathways Regulating LA/LSCC

To explore the potential influence of PPARD on LA/LSCC and improve our understanding of the underlying mechanisms, we conducted a large-scale literature data mining, based on which we built molecular pathways connecting PPARD and LA/LSCC. Specifically, we composed the pathways driven by LA/LSCC influencing the expression and activity of PPARD and the pathways driven by PPARD influencing the pathological development of LA and LSCC. The literature data mining was performed within the Pathway Studio (http://www.pathwaystudio.com) environment, which houses over 24 million PubMed abstracts and over 3.5 million Elsevier and third-party full-text papers. We initially identified the genes and functional classes that are downstream targets of PPARD and upstream regulators of LA/LSCC, manually reviewing the references and related sentences for quality control of each relationship identified. Relationships with no polarity or indirectly related to the activity of PPARD or human LA/LSCC were removed. The remaining relationships were used to construct the network describing the possible molecular pathways driven by PPARD to influence the pathological development of LA/LSCC.

### 2.4. Pathway Enrichment Analysis for LA/LSCC Regulators Driven by PPARD

To explore the functional profile of the PPARD-driven LA/LSCC regulators, we conducted a pathway enrichment analysis (PEA) using Pathway Studio. The input was the regulatory genes of LA/LSCC driven by PPARD. The background pathway database was Pathway Studio and Gene Ontology (GO) terms. These pathways/GO terms satisfy the false detective ratio analysis (*q* = 0.005) and also demonstrate an overlap of no less than 5% criteria.

## 3. Results

### 3.1. Expression of *PPARD* in LA/LSCC Datasets

**Figure 1:**
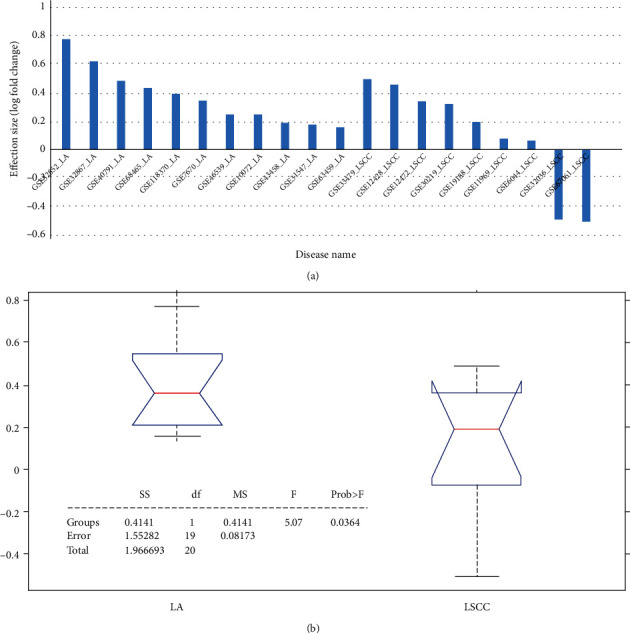
Expression of *PPARD* in 11 LA datasets and nine LSCC datasets: (a) bar plot of the expression log-fold change of PPARD; (b) boxplot of one-way ANOVA results.

A total of 20 lung cancer datasets qualified the inclusion criteria (Table 1). As shown in Figure 1, our results confirm that *PPARD* expression is increased in lung cancer, as its elevated levels were detected in all 11 LA datasets and seven out of 9 LSCC cases with log-fold changes 0.37 ± 0.20 and 0.10 ± 0.37 for LA and LSCC, respectively. Our findings are consistent with those reported previously. For the detailed results of the *PPARD* expression analysis, please refer to Supplementary Data (Expression Analysis) (available here).

In LA samples, average levels of PPARD expression were higher than those observed in LA (*p* < 0.036), Figure 1(b). The significant outliers of the PPARD expression were from datasets GSE32036 and GSE6706 which were displaying an opposite trend, which was driven by multiple outliers.

Due to a lack of relevant clinical information, we cannot determine the specific reason for the downregulation of PPARD expressions in these two datasets. However, we noted that the sample sources of dataset GSE32036 were NSCLC/SCLC cell lines using either Illumina HumanWG-6 V3 or HumanHT-12 V4. In dataset GSE67061, the PPARD expression profile was compared between LSCC lung tissue and normal airway epithelium cells. In comparison, most of the other datasets were comparing expression profiles acquired from the same source (e.g., disease/normal lung/bronchus of human). Therefore, we doubt the sample source could be a possible reason impacting the expression of PPARD in LSCC that needs further study.

### 3.2. LA/LSCC-Driven Pathways Regulating PPARD

**Figure 2:**
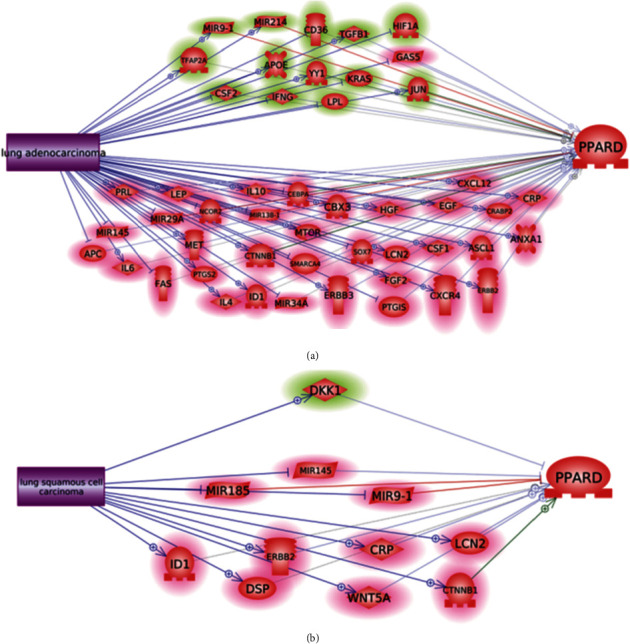
Pathways driven by LA/LSCC influencing the expression and activity of PPARD: (a) LA-driven pathway; (b) LSCC-driven pathway.

To better understand the influence of LA/LSCC on the expression of PPARD, we conducted another literature-based pathway analysis, as shown in Figure 2. These pathways showed that LSCC demonstrated an overwhelming promotion effect on the expression of PPARD through the regulation of 10 out of 11 PPARD upstream regulators (highlighted in red in Figure 2(b). In contrast, LA presented a more complex effect on PPARD. Specifically, LA could exert a positive influence on PPARD through 38 out of 49 PPARD upstream regulators, while LA may also inhibit PPARD through the regulation of 11 molecules.

### 3.3. MLR Results

Figure 1 presents the expression of PPARD variables among different studies. MLR results showed that the population region (country) of the samples could be a significant influential factor. In contrast, the sample size or the date of the sample profile collection has no significant effect (Table 2).

### 3.4. PPARD-Driven Pathways Affecting LA/LSCC

**Figure 3:**
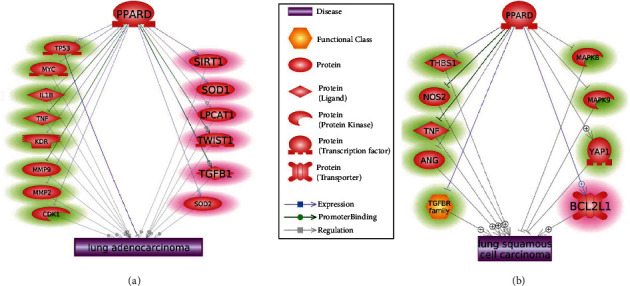
Pathways driven by PPARD influencing the pathological development of LA and LSCC: (a) pathways connecting PPARD and LA; (b) pathways connecting PPARD and LSCC.

As shown in Figure 3, PPARD modulates multiple regulators of LA and LSCC to exert influence on the etiology and development of LA and LSCC. Specifically, PPARD inhibits seven LSCC promoters (THBS1, NOS2, TNF, ANG, MAPK8, MAPK9, and TGFBR family) and seven LA promoters (MYC, IL1B, TNF, KDR, MMP9, MMP2, and CDK1). In addition, PPARD activates one LSCC inhibitor (YAP1) and one LA inhibitor (TP53). PPARD drove these molecules to inhibit the pathological development and progression of LA/LSCC. These entities are highlighted in green in Figure 3.

Several LA/LSCC promoters that could be activated by PPARD were identified, including SIRT1, SOD1, LPCAT1, TWIST1, TGFB1, and SOD2 for LA and BCL2L1 for LSCC. Activation of these promoters could cause adverse effects that PPARD could exert on LA/LSCC. These molecules are highlighted in red in Figure 3. As a side note, PPARD drove different groups of regulators to influence LA and LSCC, with only one joint promoter (TNF).

### 3.5. PEA Results for LA/LSCC Regulators Driven by PPARD

**Figure 4:**
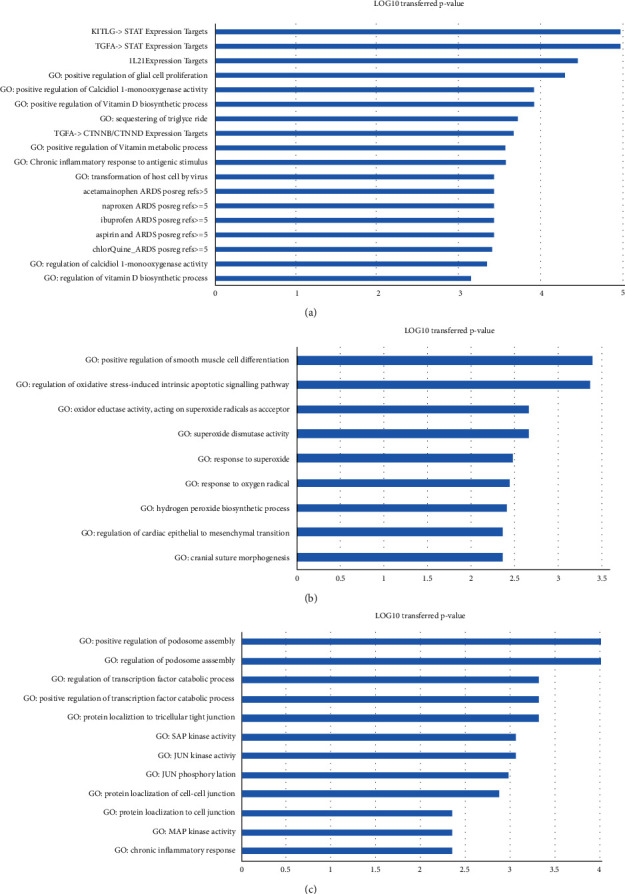
Pathway enriched analysis results: (a) the PEA results for eight PPARD-driven molecules to inhibit LA; (b) the PEA results for six PPARD-driven molecules to promote LA; (c) the PEA results for PPARD-driven LSCC regulators to inhibit LSCC.

To explore the functional profile of the PPARD-driven molecules that influence LA/LSCC, we performed three PEAS and presented the results in Figure 4. Detailed information of all the PEA results is provided in Supplementary Data: PEA4LA_Good, PEA4LA_Bad, and PEA4LSCC_Good, respectively (available here).

In Figure 4(a), we presented the top pathways/GO terms enriched by the molecules driven by PPARD (Figure 3(a)) to inhibit LA. Interestingly, besides the glial cell proliferation pathway, we also identified multiple vitamin D biosynthetic processes and regulation of calcidiol 1-monooxygenase activity-related pathways. A previous study showed that glial cell proliferation could promote the tumor cell growth of LA [11]. Moreover, vitamin D supplementation has been suggested as an approach to prevent lung cancer progression [12]. Regulation of these pathways may be part of the mechanism that PPARD may help to inhibit the progress of LA. In Figure 4(b), we presented the top pathways/GO terms enriched by the molecules driven by PPARD (Figure 3(a)) that could promote LA. Interestingly, most of these GO terms were related to superoxide, which is associated with tumor progression and migration in AFG1-induced LA [13, 14]. In Figure 4(c), we presented the pathways/GO terms enriched by the PPARD-modulated molecules that inhibit LSCC. The top two PEA results were related to podosome assembly, which has been shown to decrease the invasion and migration capabilities of LA cells [15]. The next two top GO terms were related to the transcription factor catabolic process, which has been shown to play vital roles in the development of human LA and LSCC [16, 17].

## 4. Discussion

Previous studies showed that gene PPARD demonstrated increased expression in patients with lung cancer [5, 8, 9]. However, there is a lack of discussion explaining PPARD overexpression in the etiology and development of lung cancer. In this study, we first explored the expression levels of PPARD in LA and LSCC, which account for about 70% of all lung cancer cases [1]; then, we employed a literature-based pathway analysis to explore the potential role of PPARD in LA/LSCC. Our results confirmed the overexpression of PPARD in most LA and LSCC cases. However, pathway analysis showed that the overexpression of PPARD might play a mixed role in the pathological development and progress of LA/LSCC.

Expression data analysis showed that PPARD demonstrated increased expression in 18 out of the 20 LA/LSCC independent datasets (Figure 1), which supported the previous finding that PPARD was overexpressed in the majority of lung cancers [5]. The two datasets, both were LSCC studies that presented decreased expression of PPARD, may be related to the specific drugs the patients were taking. Specifically, gene expression profiles in GSE32036 were collected from cell lines instead of patient tissues. For patients in GSE67061, the PPARD expression profile was compared between LSCC lung tissue and normal airway epithelium cells [18]. However, due to a lack of relevant information, other factors that influence the expression of PPARD in the case of LA/LSCC should be studied. The complexity of the influence of LA and LSCC on PPARD was also demonstrated through the pathways in Figures 2(a) and 2(b), respectively. The upstream regulators of PPARD could be promoted or inhibited by LA and LSCC, through which both a negative and positive influence on the expression of PPARD could occur, which may partially explain the between-dataset expression variation of PPARD.

MLR results showed that the population region (country) of the samples could be a significant, influential factor (*p* value = 0.015; see Table 2). The sample size or the date of the sample profile collection did show a notable influence on the PPARD expression among the 20 LA/LSCC datasets. Moreover, ANOVA results showed that PPARD was more overexpressed in LA than in LSCC (Figure 1(b)). Even without the influence of two outliers (GSE32036 and GSE67061), the overall PPARD expression in LSCC was still lower than that in LA (LFC = 0.270.17 and 0.370.20 for LSCC and LA, respectively). This may indicate a more prominent influence of PPARD on LA than on LSCC.

As shown in Figure 3, PPARD modulates multiple regulators of LA and LSCC to exert influence on the etiology and development of LA and LSCC. Specifically, PPARD inhibits seven LSCC promoters (THBS1, NOS2, TNF, ANG, MAPK8, MAPK9, and TGFBR family) and seven LA promoters (MYC, IL1B, TNF, KDR, MMP9, MMP2, and CDK1). For instance, THBS1 was found to promote tumorigenesis and invasion in LSCC [19], and macrophage inducible nitric oxide synthase (NOS2) promotes the initiation of LSCC [20]. Also, TNF has been shown to stimulate both LA and LSCC cells in mouse models [21]. We presented the detailed information of other LA/LSCC promoters in Supplementary Data: LSCC_PPARD Pathway and LA_PPARD Pathway (available here). By inhibiting these LA/LSCC promoters [22–24], the overexpression of PPARD could inhibit the initiation and progress of lung tumor cells.

In addition, PPARD activates one LSCC inhibitor (YAP1) and one LA inhibitor (TP53), which may be another mechanism of how PPARD could inhibit the pathological development of LA/LSCC. PPARD has also been shown to interact with YAP1 to promote gastric tumorigenesis [25], and YAP1 was suggested as a suppresser of LSCC through the reactivation of oxygen species accumulation [26]. Moreover, PPARD-induced P53 activation [27] has been shown to play a protective role in human LA [28, 29].

In addition, PPARD could also negatively influence the pathological development of LA/LSCC through the upregulation of the promoters of LA/LSCC. As shown in Figure 3(a), PPARD could activate six LA promoters and thereby facilitate the initiation and progression of LA. For example, PPARD agonist has been shown to increase SIRT1 protein levels [30], which is a tumor promoter in LA [31]. Activation of PPARD has also been associated with the increased expression of both SOD1 and SOD2 [32, 33], which are linked with tumor progression and migration in AFG1-induced LA [34]. Moreover, Weeden et al.'s study showed that it is necessary to inhibit both BCL2L1 and MCL1 [34] to induce tumor regression in LA sensitive to FGFR inhibition. However, PPARD has been shown to increase the expression of BCL2L1 [8], suggesting a PPARD-LSCC-promoting mechanism. For more of these vicious roles that PPARD could play in LA/LSCC, please see Supplementary Data: Ref4_LA_PPARD_Pathway and Ref4_LSCC_PPARD_Pathway (available here).

To note, PPARD drove different groups of regulators to influence LA and LSCC, with only one common promoter (TNF). This finding indicated that PPARD could exert influence on LA and LSCC through a different mechanism. PEA results showed that, on the one hand, LA might play a protective role against LA progression through the regulation of vitamin D biosynthetic process and glial cell proliferation [11, 12]. On the other hand, PPARD may modulate the response to oxygen radicals and superoxide to promote the development of LA [13, 14]. Moreover, PEA results also suggested that the mechanism that PPARD influences LSCC might be related to podosome assembly and the transcription factor catabolic process [15–17]. We provided the details of the pathways in Figure 4 and in Supplementary Data: PEA4LA_Good, PEA4LA_Bad, and PEA4LSCC_Good (available here).

Our study guaranteed several future works. First, in this study, we only used expression array data to study the expression of PPARD in LA/LSCC. Data of other modalities, including RNA sequencing data, should be used to validate this study's results. Second, besides sample population region, sample size, and sample profile collection date, more factors influencing the PPARD expression (e.g., age and gender) should be tested when data are available.

## 5. Conclusion

Our results confirmed the increased gene expression of PPARD in the majority of cases of LA/LSCC. However, our pathway analysis indicated a mixed effect of the overexpression of PPARD on the pathological development and progression of LA and LSCC. The PPARD-driven pathways identified in our study may provide new insights into the understanding of the roles that PPARD plays in lung cancer.

## Figures and Tables

**Table 1:** Key descriptors of 20 LA/LSCC RNA expression datasets selected for this study.

GEO ID	Disease name	*N* controls	*N* cases	Sampled population (country)	Time factor	Sample source
GSE7670	LA	28	27	Taiwan	13	Adenocarcinoma/normal lung
GSE68465	LA	4	443	USA	5	Adenocarcinoma/normal lung
GSE67061	LSCC	8	69	China	3	LSCC/normal airway epithelium cells
GSE63459	LA	32	33	USA	5	Adenocarcinoma/normal lung
GSE6044	LSCC	5	14	Germany	13	LSCC/normal lung
GSE51852	LA	4	49	Japan	6	Adenocarcinoma/normal lung
GSE46539	LA	92	92	Taiwan	4	Adenocarcinoma/normal lung
GSE43458	LA	30	80	USA	7	Adenocarcinoma/normal lung
GSE40791	LA	90	94	USA	7	Adenocarcinoma/normal lung
GSE33479	LSCC	27	14	USA	5	LSCC/normal bronchus
GSE32867	LA	58	58	USA	8	Adenocarcinoma/normal lung
GSE32036	LSCC	59	12	USA	7	NSCLC/SCLC cell lines
GSE31547	LA	20	30	USA	9	Adenocarcinoma/normal lung
GSE30219	LSCC	14	61	France	5	LSCC/normal lung
GSE19188	LSCC	65	27	Netherlands	9	LSCC/normal lung
GSE12472	LSCC	28	35	Netherlands	10	COPD bronchus/LSCC lung
GSE12428	LSCC	28	34	Netherlands	11	Normal bronchial/LSCC lung
GSE11969	LSCC	5	35	Japan	10	Adenocarcinoma/normal lung
GSE118370	LA	6	6	China	1	Adenocarcinoma/normal lung
GSE10072	LA	49	58	USA	12	Adenocarcinoma/normal lung

Note: “time factor” refers to the age of the dataset, which is defined by the current year–the publication year of the dataset.

**Table 2:** Multiple linear regression analysis of three potential factors for PPARD expression in LA/LSCC.

	Sample #	Country	Study age
Beta	-1.97*E*-05	0.077	5.10*E*-4
Low limit	-1.63*E*-3	-0.01	-0.044
Up limit	1.59*E*-3	0.16	0.044
*p* value	0.51	0.015	0.49

## Data Availability

The data in our study are available from the corresponding author upon reasonable request.

## Supplementary Materials

Supplementary Materials The Supplementary Data is a multiworksheet Excel file that contains additional results described as follows: (1) Expression Analysis: the detailed results of the PPARD expression analysis in 11 LA datasets and 9 LSCC datasets that met the inclusion criteria; (2) Ref4_LSCC_PPARD Pathway: information regarding LSCC_PPARD Pathway was presented, including the type of the relationship, supporting references, and related sentences from the references where the relationship has been identified; (3) Ref4_LA_PPARD_Pathway: information regarding LA_PPARD Pathway was presented, including the type of the relationship, supporting references, and related sentences from the references where the relationship has been identified; (4) PEA4LA_Good: detailed information on all the PEA results that may inhibit the progress of LA; (5) PEA4LA_Bad: detailed information on all the PEA results that may promote the progress of LA; and (6) PEA4LSCC_Good: detailed information on all the PEA results that may inhibit the progress of LSCC. The excel file is online available at http://www.gousinfo.com/database/Data_Genetic/PPARD_Lung Cancer.xlsx.
