# Supplementary material for: Increased PPARD Expression May Play a Protective Role in Human Lung Adenocarcinoma and Squamous Cell Carcinoma
Source: PPAR Res. 2022 Mar 15;2022:9414524. doi: 10.1155/2022/9414524 (PMC8941584; doi:10.1155/2022/9414524)
Supplement: Supplementary Materials — The Supplementary Data is a multiworksheet Excel file that contains additional results described as follows: (1) Expression Analysis: the detailed results of the PPARD expression analysis in 11 LA datasets and 9 LSCC datasets that met the inclusion criteria; (2) Ref4_LSCC_PPARD Pathway: information regarding LSCC_PPARD Pathway was presented, including the type of the relationship, supporting references, and related sentences from the references where the relationship has been identified; (3) Ref4_LA_PPARD_Pathway: information regarding LA_PPARD Pathway was presented, including the type of the relationship, supporting references, and related sentences from the references where the relationship has been identified; (4) PEA4LA_Good: detailed information on all the PEA results that may inhibit the progress of LA; (5) PEA4LA_Bad: detailed information on all the PEA results that may promote the progress of LA; and (6) PEA4LSCC_Good: detailed information on all the PEA results that may inhibit the progress of LSCC. The excel file is online available at http://www.gousinfo.com/database/Data_Genetic/PPARD_Lung Cancer.xlsx. [file 9414524.f1.pdf]

Supplementary Data

The Supplementary Data is a multi-worksheet excel file that contains additional results described as follows.

1) Expression Analysis: The detailed results of the PPARD expression analysis in 11 LA datasets and 9 LSCC datasets that met the inclusion criteria.
2) Ref4_LSCC_PPARD Pathway: information regarding LSCC_PPARD Pathway was presented, including the type of the relationship, supporting references, and related sentences from the references where the relationship has been identified.
3) Ref4_LA_PPARD_Pathway: information regarding LA_PPARD Pathway was presented, including the type of the relationship, supporting references, and related sentences from the references where the relationship has been identified.
4) PEA4LA_Good: detailed information on all the PEA results that may inhibit the progress of LA
5) PEA4LA_Bad: detailed information on all the PEA results that may promote the progress of LA.
6) PEA4LSCC_Good: detailed information on all the PEA results that may inhibit the progress of LSCC

The excel file is online available at:
www.gousinfo.com/database/Data_Genetic/PPARD_Lung_Cancer.xlsx
